# Use of complementary and alternative medicine within Norwegian hospitals

**DOI:** 10.1186/s12906-015-0782-5

**Published:** 2015-08-13

**Authors:** R Jacobsen, V. M. Fønnebø, N. Foss, A. E. Kristoffersen

**Affiliations:** The National Research Center in Complementary and Alternative Medicine (NAFKAM), Department of Community Medicine, Faculty of Health Sciences, UiT The Arctic University of Norway, Tromsø, Norway

**Keywords:** CAM, Hospitals, Norway, Trends, Complementary therapies, Alternative medicine

## Abstract

**Background:**

Over the recent decades complementary and alternative medicine (CAM) use within and outside of the public health care system in Norway has increased. The aim of this study is to describe to what extent CAM is offered in Norwegian hospitals in 2013 and investigate possible changes since 2008.

**Methods:**

In January 2013 a one-page questionnaire was sent to the medical director of all included hospitals (*n* = 80). He/she was asked to report whether or not one or more specific CAM therapies were offered in the hospital. Fifty-nine (73.8 %) hospitals responded and form the basis for the analyses.

**Results:**

CAM was offered in 64.4 % of the responding hospitals. No major differences were found between public and private, or between somatic and psychiatric, hospitals. Acupuncture was the most frequent CAM method offered, followed by art- and expression therapy and massage.

The proportion of hospitals offering CAM has increased from 50.5 % in 2008 to 64.4 % in 2013 (*p* = 0.089). The largest increase was found in psychiatric hospitals where 76.5 % of hospitals offered CAM in 2013 compared to 28.6 % in 2008 (*p* = 0.003). A small decrease was found in the proportion of hospitals offering acupuncture between 2008 (41.4 %) and 2013 (37.3 %).

**Conclusions:**

A majority of Norwegian hospitals offer some sort of CAM. The largest increase since 2008 was found in psychiatric hospitals. Psychiatric hospitals seem to have established a practice of offering CAM to their patients similar to the practice in somatic hospitals. This could indicate a shift in the attitude with regard to CAM in psychiatric hospitals.

## Background

Over the recent decades there has been a substantial increase in the use of complementary and alternative medicine (CAM) within and outside of the health care system. Several international studies have demonstrated this trend both in the general population, as well as in different patients groups [1–7].

A recent systematic review of population use of CAM found that the prevalence of CAM use during the preceding 12 months ranged from 10 to 76 % [7]. The prevalence of ever users of CAM in the Scandinavian countries ranged from 34 to 49 % [6]. A recent Norwegian survey shows that 45 % of the respondents had used CAM within the last 12 months. The use of CAM seemed to be unchanged since 2007 [8]. Massage was the most commonly used CAM method, followed by acupuncture [4, 8, 9].

A survey regarding attitude towards, and use of, CAM among different occupational groups within hospitals in the north of Norway, showed a more positive attitude towards CAM among office staff and nurses (71–72 %) than among medical doctors (16 %). Nurses, as well as young females in all occupational groups were most positive to the use of CAM. They were also more interested in knowledge and information about CAM [10].

As the request for CAM continues to increase, health care systems in some countries seem to integrate these therapies into conventional medical care [9, 11].

Acupuncture has been an integrated part of the clinical practice of Norwegian general practitioners (GPs) who have undergone acupuncture training. Of 111 GPs with acupuncture training responding to a questionnaire, 60 % used acupuncture to treat patients. Fifty-two per cent used acupuncture in more than 5 % of their consultations. Acupuncture was most often used to treat musculoskeletal pain, migraine and tension headache, but was also used in nausea, indigestion, allergies, asthma and sleeping disorders. For the most common disease groups, positive effect was reported in 3 out of 4 patients [12].

In many Western countries, the use of CAM is well integrated into the general health care system. One example is the state of Washington in the United States, where 86 % of hospices offered CAM to their patients in 2006. The therapies were offered by volunteers and were not covered under hospice benefits [13]. Interest from the patients, and improvement in quality of life in end-of-life care, were the main reasons for the high number of hospices offering CAM [13].

The Royal London Hospital for Integrated Medicine (RLHIM) has offered CAM for more than 160 years, and is the largest public hospital in England offering CAM. The hospital has been acknowledged by the National Health Service since 1948, and was in 2002 integrated as a part of The University College London Hospitals [14]. The treatment of patients takes place in outpatient clinics run by medical doctor specialists [14]. The hospital combines conventional and alternative treatment, and has a close collaboration with other university hospitals. In Norway, on the other hand, there are no public outpatient clinics offering mainly CAM [14]. However, several Norwegian public and private hospitals seem to integrate use of CAM, especially acupuncture, during delivery [15]. Patient demand is the main reason why hospitals decide to provide a specific type of CAM therapy [11].

The provision of CAM in Norwegian hospitals has been studied twice, in 2001 and 2008. From 2001 to 2008 the proportion of Norwegian hospitals offering CAM increased from 25 % to 50.5 % [15, 16]. Acupuncture was the therapy most frequently offered [15, 16]. The aim of this study is to (1) describe to what extent CAM is offered in Norwegian hospitals in 2013 and (2) investigate possible changes since 2008.

## Methods

### Organisation of the hospitals

In Norway the public secondary and tertiary health care service is organized within four regional health authorities. These regional health authorities are responsible for the health care service in a given part of the country. Each regional health authority is subdivided into smaller local health authorities, which again are responsible for one or more smaller hospitals, with both somatic and psychiatric departments. The regional health authorities are funded by the government, and are part of the public health service. During the first decade of the 21^st^ century there has been a merger of several small public hospitals to fewer, larger hospitals. The total number of Norwegian public hospitals has thereby decreased. In addition to public hospitals, there are several smaller private hospitals, both somatic and psychiatric. Some of these have a funding contract with the regional health authorities and are therefore accessible to the public in the same way as public hospitals (free of charge for the patient). Other private hospitals operate independently of the regional health authorities and require full payment directly from the patients.

Several public psychiatric institutions were converted into district psychiatric centres (DPS) in 2010, or closed down. Public psychiatric care was in some cases moved to separate departments within somatic hospitals. Somatic and psychiatric departments can therefore be located within the same public hospital. In this survey nine of the invited public hospitals were registered as psychiatric hospitals, as these only have psychiatric units. The rest of the public hospitals are registered as somatic hospitals, even though several of these also include psychiatric units.

This study includes:

1. All local health authorities in Norway (*n* = 21) including 59 public hospitals (50 somatic with or without psychiatric departments and 9 psychiatric).
2. All private hospitals (6 somatic and 15 psychiatric) with more than 10 beds and a funding contract with the regional health authorities as of December 31, 2010 (*n* = 21).

Private hospitals without a funding contract with the regional health authorities were not included in this study. This was done because we wanted to limit our study to hospitals generally available to all Norwegian patients regardless of ability to pay out of pocket for the service.

### The questionnaire

In the beginning of January 2013, a one-page questionnaire was sent to all local health authorities and private hospitals included in the study. The hospitals had been contacted by phone in advance, to provide the name of the most relevant person to receive the questionnaire, usually the person clinically responsible for medical services. All 21 local health authorities and every private hospital included in the study received one envelope. A maximum of two e-mail reminders were sent if the questionnaire was not returned.

In the envelope sent to the health authorities there was one numbered questionnaire for each hospital within the health authority. The person clinically responsible was either asked to answer on behalf of all the hospitals or distribute the questionnaires to relevant recipients. The person clinically responsible was asked whether or not CAM therapies were offered at the hospital. He or she was asked to tick one or more of seven categories: Acupuncture, massage, psychotherapy (not psychologist services), art- and expression therapy, alternative diet, other CAM therapy (Please specify) or no CAM offered. None of the therapies were described further, leaving it to the person clinically responsible to decide what to consider as for example an alternative diet or art- and expression therapy (will include music therapy). Psychotherapy (not psychologist services) will typically include techniques like gestalt therapy that is mainly practised outside the health care system in Norway. For each reported therapy, the name of a contact person was asked for.

The specified CAM treatments in the questionnaire all fall within the Norwegian legal definition of CAM: “health-related treatment which is practiced outside the established health services and which is not practiced by authorized health personnel. However, treatment practiced within the scope of the established health services or by authorized health personnel is also covered by the term alternative treatment when the methods employed essentially are used outside the established health services” [17].

**Fig. 1:**
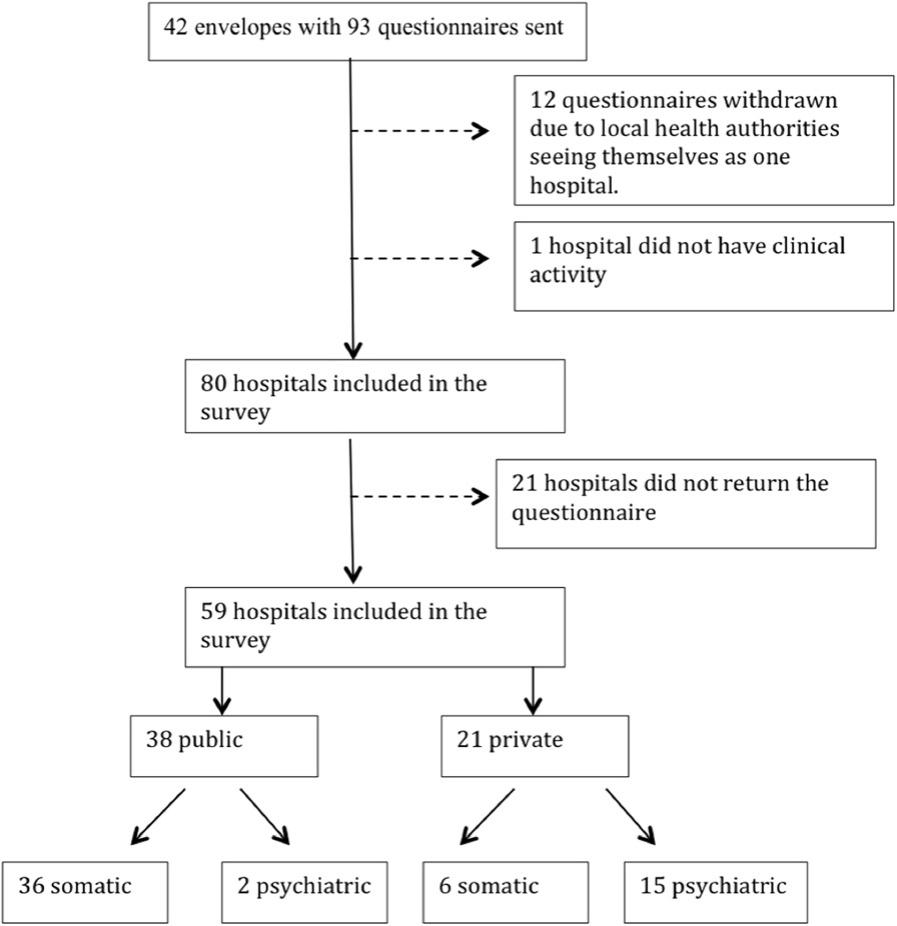
Hospitals participating in the survey

Forty-nine out of 93 questionnaires were filled out and returned after the first dispatch. Four local health authorities returned only one questionnaire for the local health authority as a whole since they saw themselves as only one hospital. Twelve questionnaires were thereby withdrawn from the survey. One hospital reported no clinical activity, and was therefore excluded, leaving 80 valid questionnaires. Some hospitals had made copies of their questionnaire and returned more than one questionnaire with the same registration number. If at least one of the returned questionnaires from the same hospital indicated CAM modalities in use, the hospital was categorized as offering CAM. Nine hospitals responded after a first reminder, one after the second reminder. The inclusion process of the hospitals is shown in Fig. 1.

### Response rate in public and private hospitals

The response rate was higher in private (100 %) than public hospitals (64.4 %), and higher in somatic (75 %) than psychiatric hospitals (70.8 %). Public psychiatric hospitals had the lowest response rate (22.2 %) (Table 1).

**Table 1:** Response rates

	Invited	Responded	%
Total	*n* = 80	*n* = 59	73.8
Somatic	56	42	75.0
Psychiatric	24	17	70.8
Public	59	38	64.4
Somatic	50	36	72.0
Psychiatric	9	2	22.2
Private	21	21	100
Somatic	6	6	100
Psychiatric	15	15	100

### Telephone interviews

To further understand the development of CAM offered within Norwegian hospitals, and to confirm the increase of psychiatric hospitals offering CAM, a semi structured telephone interview was conducted with person clinically responsible at four public psychiatric hospitals that did not return the self-administered questionnaire.

### Statistical methods

All data were analysed using SPSS version 21.0. Differences between groups were analysed using Pearson’s chi-square tests and Fisher’s exact test.

### Ethical approval

Due to lack of human information or material at the individual level, ethical approval was due to the guidelines, not required.

## Results

### Norwegian hospitals offering CAM

A majority of the hospitals (64.4 %) reported to offer one or more CAM therapies to their patients. This proportion was similar in public (63.2 %) and private hospitals (66.7 %, *p* = 0.788). Slightly more psychiatric (76.5 %) than somatic hospitals (59.5 %) reported to offer CAM (*p* = 0.218, Table 2). The highest proportion was found in private psychiatric hospitals (80 %), while the lowest was found in private somatic hospitals (33.3 %, *p* = 0.064, Table 3).

**Table 2:** Hospitals offering CAM in Norway

Total n	Public n	Private n		Somatic n	Psychiatric n	
n (%)	n (%)	n (%)	*p*=	n (%)	n (%)	*p*=
38 (64.4)	24 (63.2)	14 (66.7)	0.788*	25 (59.5)	13 (76.5)	0.218*

*Pearson Chi-Square test

**Table 3:** CAM use within Norwegian hospitals

	Total	Offer CAM	%	
	*n* = 59	*n* = 38	64.4	*p*-value
Public	38			
Somatic^a^	36	23	63.9	
Psychiatric	2	1	50.0	*p* = 0.607*
Private	21			
Somatic	6	2	33.3	
Psychiatric	15	12	80.0	*p* = 0.064*

*Fisher’s Exact Test

^a^ Several of the public somatic hospitals also include psychiatric units

### CAM offered in Norwegian hospitals in 2013 compared to 2008

No major differences were found in the proportion of somatic hospitals offering CAM in 2013 (59.5 %) compared to 2008 (56.4 %, *p* = 0.742). In psychiatric hospitals, on the other hand, an increase from 28.6 % in 2008 to 76.5 % in 2013 (*p* = 0.003, Table 4) was found. In 2008 the highest proportion of hospitals offering CAM was public somatic hospitals (58 %) [15], while private psychiatric hospitals had the highest proportion in 2013 (80 %, Table 3).

**Table 4:** CAM use in somatic and psychiatric hospitals in 2008 and 2013

		Offer CAM to patients		
		2008	2013	
Somatic	n	44	25	
	%	56.4	59.5	*p* = 0.742*
Psychiatric	n	6	13	
	%	28.6	76.5	*p* = 0.003*
Total	n	50	38	
	%	50.5	64.4	*p* = 0.089*

*Pearson Chi-Square test

No major differences were found in proportion of public hospitals offering CAM in 2013 (63.2 %) compared to 2008 (61.2 %, *p* = 0.842). In private hospitals, on the other hand, an increase from 28.1 % in 2008 to 66.7 % in 2013 (*p* = 0.006, Table 5) was found.

**Table 5:** CAM use in public and private hospitals in 2008 and 2013

		Offer CAM to patients		
		2008	2013	*p*-value
Public	n	41	24	
	%	61.2	63.2	0.842*
Private	n	9	14	
	%	28.1	66.7	0.006*

*Pearson Chi-Square test

### Therapies offered

In 2008, 13.1 % (*n* = 13) of the hospitals offered more than one CAM therapy, compared to 25.9 % (*n* = 15) in 2013. Among the hospitals offering CAM, the mean number of therapies offered was 1.5 in 2008 and 2.1 in 2013.

All CAM modalities were offered in more hospitals in 2013 than in 2008, except for acupuncture, which had decreased from 41.4 % (*n* = 41) in 2008 to 37.3 % (*n* = 22) in 2013. Acupuncture was, however, still the most commonly offered therapy, followed by art-and expression therapy (25.4 %), massage (15.3 %) and alternative diet (8.5 %). Only 5.1 % of the hospitals reported to offer psychotherapy (not psychologist services). Other types of CAM were also offered by 27.1 % of the hospitals, such as music therapy, gestalt therapy, hypnosis, acupressure, yoga and mindfulness. Music therapy was the most frequent therapy in this category, offered by 13.6 % (*n* = 8) of the hospitals. The therapies which had increased the most from 2008 were art- and expression therapy and the category “other therapies” (Table 6).

**Table 6:** CAM modalities offered in 2008 and 2013

	2008	2013	
	n (%)	n (%)	*p*-value
Acupuncture	41 (41.4)	22 (37.3)	0.608*
Art- and expression therapy	4 (4.0)	15 (25.4)	0.000*
Massage	8 (8.1)	9 (15.3)	0.159*
Alternative diet	7 (7.1)	5 (8.5)	0.763**
Psychotherapy (not psychologist services)	1 (1.0)	3 (5.1)	0.147**
Other	7 (7.1)	16 (27.1)	0.010*
No CAM offered	49 (49.5)	21 (35.6)	0.089*

*Pearson Chi-Square test, **Fisher’s Exact Test

Within psychiatric units, art- and expression therapy and music therapy were the most commonly offered therapies (52.9 %, *n* = 8) in 2013, followed by acupuncture (17.6 %, *n* = 3). In 2008 massage (14.3 %, *n* = 3) dominated followed by acupuncture and art- and expression therapy (both 9.5 %, *n* = 3).

## Discussion

In 2013, more than 64 % of Norwegian hospitals offered CAM to their patients, with private psychiatric hospitals reporting the highest proportion (80 %).

### Bias considerations

The response rate of 73.8 % (Table 1) is somewhat lower than in 2001 (94 %) and 2008 (85 %). The lower response rate in this study might be due to a different organization of the public hospitals in 2013, where one person has the clinical responsibility for several local hospitals, with different locations and with several units. This could also have lead to a lower overview of CAM modalities in use. The presumed lack of overview of therapy methods offered could have resulted in under-reporting of CAM on offer.

The reminders were in this survey sent by e-mail instead of by ordinary postal service. This could possibly also have influenced the response rate, because it probably might be easier to oversee an e-mail than an envelope in the mail.

The low response rate in public psychiatric hospitals (22.2 %), where only two out of nine responded, makes the results for psychiatric hospitals most accurate for private hospitals. A telephone interview with four of the seven non-responding public psychiatric hospitals, suggested that also these offered CAM to their patients. The low response rate in this group is therefore not likely to have led to an overestimation of hospitals offering CAM.

Integration of CAM within the conventional health service is associated with different perspectives, attitudes and points of view. Due to this we cannot exclude the possibility that the hospitals most positive to CAM answered more frequently than those who are more sceptical.

The comparison of CAM offered within somatic and psychiatric hospitals might be inaccurate, because several public somatic hospitals also include psychiatric units, and then again offer CAM within these units. However, most of the public somatic hospitals that reported offering CAM within psychiatric units, also reported offering CAM within their somatic units. Even though they also offer CAM within psychiatric units we have registered the hospitals as somatic in our analyses.

The higher number of psychiatric hospitals reporting to offer CAM in 2013 compared to 2008, might partly be due to changes in the questionnaire. While the 2008 questionnaire started with “no CAM offered”, the 2013 questionnaire ended with this option after a list of potential CAM therapies. Also the fact that the most offered therapy, art- and expression therapy, was specified only in 2013 might have influenced the reporting of CAM therapies offered. An interview with three hospitals reporting no CAM in 2008 while reporting to offer CAM in 2013 supports this possibility. They all confirmed that they before 2008 offered CAM therapies not specified in the 2008 questionnaire. The reason why they did not themselves add these therapies in 2008, was that they did not consider them to be CAM. The substantial increase between 2008 and 2013 could therefore partly be a result of under-reporting in 2008.

Our findings of slightly more hospitals offering CAM in 2013 compared to 2008 could be the result of a lower general response rate combined with a higher number of responses from hospitals offering CAM. If all the non-responders are classified as not offering CAM, the number of Norwegian hospitals offering CAM would have been 47.5 % (a decrease from 2008 by 3 %). If all the non-responders, on the other hand, are classified as offering CAM the number of Norwegian hospitals offering CAM would have been 73.6 % (an increase of 23.1 %).

### Trends in CAM offered within Norwegian hospitals

The increase in hospitals offering CAM from 2001 to 2013 might be caused by an increase of CAM use in the general population during the beginning of the 21^st^ century [1, 4, 5, 9]. Health care workers’ general attitude towards CAM have also become more positive, and more of them wish to include CAM into health care and hospitals [9, 10]. Health care workers with training within CAM therapies seem to be allowed to practice these therapies in the hospital [10, 18–20]. The lower increase in somatic units might be due to the already high proportion already offering CAM in this hospital group. The substantial increase in private psychiatric units offering CAM might be due to several factors; more staff with CAM training, more research supporting CAM treatment of mental disorders [21] and request from patients for a non-pharmaceutical treatment option. The increase could also be lower than indicated because three hospitals who did not report CAM use in 2008, in telephone interviews in 2013 stated that CAM also was offered before 2008.

The decrease in the proportion of Norwegian hospitals offering acupuncture is in accordance with findings of a decreased general use of acupuncture in the Norwegian population [22]. One of the reasons for this might be a popular 2012 television series in the main Norwegian TV channel criticizing CAM in general and the level of documented effect of acupuncture more specifically. The programs were heavily debated in the media, and several CAM providers reported a significant decrease in patients seeking their services. This public debate could have lead to: 1. An *underreporting* of acupuncture offered in Norwegian hospitals. 2. A real *reduction* in acupuncture offered and 3. A decreased *demand* for acupuncture.

### Comparison with other studies

Many surveys worldwide have studied CAM use among the general population and in different patient groups [2, 3, 19, 23]. Few seem to have studied CAM use provided within hospitals separately [15, 16, 24]. Our findings showed a higher proportion of hospitals offering CAM compared to Denmark (2008) and Switzerland (2005) [15, 24]. These surveys from Denmark and Switzerland were, however, published a few years ago and this could possible explain the differences found. In line with our findings, acupuncture was reported to be the most offered discipline in both Denmark and Switzerland. Other than these, no other comparable studies were found.

## Conclusion

The total number of Norwegian hospitals offering CAM has increased since 2008, and there has been an increase in use both within somatic and psychiatric, public and private hospitals. The highest increase is seen in private psychiatric hospitals. This could indicate a shift in the attitude towards CAM in psychiatric hospitals. Little is known about the extent of use within each hospital, only whether they offer CAM or not.

## Abbreviations

CAM: Complementary and Alternative Medicine
NAFKAM: National Research Centre in Complementary and Alternative Medicine
